# On Some Relations of Diseases of the Nose and Throat to Dentistry

**Published:** 1894-08

**Authors:** John W. Farlow

**Affiliations:** Boston, Mass.


### ON SOME RELATIONS OF DISEASES OF THE NOSE AND THROAT TO DENTISTRY.[1]

BY JOHN W. FARLOW, M.D., BOSTON, MASS.

It gives me great pleasure to meet, in a social way, you gentlemen of the dental profession. In our daily work, no matter how interesting or absorbing, we must certainly acknowledge that most of the time we look "down in the mouth." But to-night with our palates in good working order, we can take a more cheerful view of life. We all know that in our homes, especially if we live in

---

[1] Read before the American Academy of Dental Science, April 4, 1894.

apartment houses, we are very much interested in, and not infrequently very much annoyed by, what goes on overhead. Just so it is, or should be, of great importance to the dentist to know what is going on in the story above the mouth. The roof of the mouth is the floor of the nose, and what affects one must certainly have its influence on the other.

The mouth is for eating and speaking, and is not intended to replace the nose in breathing. The nose has for its functions to warm and moisten the inspired air, and this can be done by the mouth to but a very limited extent. When a part of the body is obliged to do what it is not adapted for, the work is usually badly done.

Let us consider for a moment what are some of the conditions of the nose which throw upon the mouth work it ought not to be called on to perform. The nose is divided into the two nostrils by the nasal septum, made up of the anterior or cartilaginous part, and the posterior or bony part. Bends, ridges, or outgrowths of this septum can so occlude one or both nostrils as to make the nose unable to fulfil its functions, and the open mouth results. As a matter of fact, it is very uncommon to find a straight septum after childhood, and great obstructions in the nostrils are very common. If the roof of the mouth is very high, it is generally the case that the nasal septum is much bent, causing a blocking of the nose to such an extent that the respiration must be carried on by the mouth. It is needless for me to call your attention to the deleterious effect on the teeth of the constant passage of air through the mouth.

On the outerside of the nostrils are the turbinated or spongy bones, which are extremely liable to hypertrophy, thus closing more or less completely the nasal passages. If this condition becomes chronic, it is a very effective means of closing the nose and hindering the entrance of air.

Nasal polypi of sufficient size to fill the nostrils are an occasional cause of mouth-breathing. The most important and, until recently, the least recognized of the diseases obstructive to nasal respiration is the one so common in childhood and early adult life, —adenoid disease, or the growth of an excessive amount of lymphoid tissue in the post-nasal space. Occurring before, or at the time of, the second dentition, by preventing the passage of the air through the nostrils it causes a narrowing and elongation of the upper jaw, with the high or V-shaped palate and a crowding together of the teeth, especially the central incisors. The upper lip is short and does not cover the teeth, and the aperture of the mouth becomes triangular. Another symptom lately spoken of is salivation. It is no wonder that such children are very prone to all sorts of displacements and tardy appearance of teeth, as well as marked tendency to decay. According to my experience, it is by all means the most rational plan to try to restore the nose to its proper function before undertaking the difficult and irrational task of bringing the jaw and teeth into their proper places while the thing which caused the deformity is still acting. It may be that I am advocating a course which will give the dentists less to do in the future, but there will certainly be fewer deformed mouths and fewer decayed and false teeth among your patients if the post-nasal spaces of your young and growing patients are freed from their obstructions.

This question of post-nasal obstruction is so important, and leads to such disastrous results with regard to the mouth and teeth when not properly recognized and treated, that it seems to me that dentists should never neglect to inform themselves in all cases of high or V-shaped palates or elongated jaws, or where the incisors overlap or the teeth very readily decay, whether there is not some obstacle to nasal respiration and consequent faulty development of the bones of the nose and mouth.

The symptoms in such cases are mouth-breathing, especially at night, short upper lip, triangular mouth, thick voice, snoring, frequent and long-continued colds in the head, occasional attacks of deafness or pain in the ear, cough, and possible lack of development. Any or all of these symptoms may be present, but the absence of some of the most prominent ones should not lead you into the possible error of therefore concluding that the post-nasal space is not considerably occluded. I have removed very large adenoid growths where many of the classical symptoms were wanting. The rhinoscopic mirror or digital examination will be requisite to make an accurate diagnosis.

In children large tonsils are nearly always accompanied with adenoid disease; and if, in looking into the throat, there are found large raised follicles on the posterior pharyngeal wall, standing out like large, red, swollen grains of tapioca, we may reasonably infer that the same condition, only much more marked, exists in the post-nasal space. The glands under the angle of the jaw are apt to be large and tender. I have thus far spoken of the bearing that various nasal diseases have on the jaws and teeth, and now I should like to say a few words about how they add greatly to the dread that some persons have of the dentist's chair. I never expect to meet in this world any one who will say that he really enjoys his seance with the dentist. It is merely a question of degree of discomfort. I think very few of you would enjoy yourselves if, while suffering from a bad cold with blocked-up nose, you had to spend an hour with your mouth open and filled with napkins, drills, or a rubber dam. You would probably write to have your appointment put off.

But persons with bent noses and large turbinated bones are in the state of being stuffed up all the time. A child who has great difficulty in breathing at night, even with the mouth wide open, who snores and kicks off all the bedclothes, cannot be expected to look on the dentist's chair as much short of an invention of the devil.

I think much can be done to bring about a more friendly relation between dentist and patient by certain simple means. If there is considerable secretion in the nose, as is apt to be the case, it is well to have the patient use an alkaline nasal spray in an atomizer. This properly used can help to clear the nose and post-nasal space. Then a weak solution of cocaine, about two or four per cent., either in water or liquid vaseline, instilled into the nose just before getting into the dentist's chair, will often reduce the swelling of the turbinated bone sufficiently to allow a free passage of the air through the nose. Cocaine used in the pharynx causes a disagreeable, choking feeling, and should be avoided.

There are many noses that weep or discharge a copious, watery mucus a good deal of the time. This is particularly the case in young, nervous women and sufferers from what is like hay-fever, except that it comes on under any excitement and not at regular seasons. A small dose of atropine, about $\frac{1}{200}$ of a grain, taken three or four hours before the visit to the dentist will often stop the leaking nose, and the handkerchief can be kept in the pocket instead of constantly at the nose.

Dr. Farlow showed a girl, thirteen years of age, whose upper jaw was very long, narrow, and high (Fig. 1). She had all the typical signs of adenoid disease,—thick voice, mouth-breathing, deafness from disease of both ears, etc. Later, he removed from her post-nasal space, by means of large forceps, the adenoid mass, which is shown here, in exact size, in Fig. 2.

